# Effects on personal control of people with psychotic vulnerability by the use of an internet-based program (PCR): a pilot study

**Published:** 2012-06-15

**Authors:** Ina Boerema, Rob de Leeuw

**Affiliations:** Trimbos, Netherlands Institute of Mental Health and Addiction, The Netherlands; University Medical Center Utrecht, The Netherlands

**Keywords:** disease management, schizophrenia, self management, internet platform

## Abstract

**Introduction:**

Starting in 2008 an internet-based platform: Personal Control and Rehabilitation (PCR) has been developed for people with psychotic vulnerability and their formal and informal caregivers and was tested in two mental health institutions in The Netherlands. The Trimbos-institute and the Julius-Centrum evaluated the development, the implementation and the preliminary effects of PCR on patients, informal caregivers and professionals.

**Aims of the study:**

It was expected that personal control and communication between patients and their (informal) caregivers will be facilitated by the use of PCR. Patients have insight in their treatment goals and medication. Communication no longer depends on the hours that the caregivers are available.

**Methods:**

The development and implementation of PCR was followed by an effect and process evaluation. The process of implementation of PCR was evaluated with interviews, validated questionnaires and focusgroup discussions. The questionnaires measure Quality of life, Empowerment, Satisfaction with care, and social functioning (patients), sense of burden (informal caregivers), job satisfaction and workload (professionals) before the implementation of PCR, three and six months later.

**Results:**

Results show that patients are satisfied with the use of PCR. They reported more insight in their treatment goals and easier communication with each other and (in)formal caregivers. Professionals are enthusiastic of the idea of an internet tool for this target group, as long as it is not a replacement for face-to-face contact. These and additional results will be presented and discussed.

**Conclusions:**

The use of PCR is appreciated by patients, although professionals and informal caregivers have to get more involved.

